# Human Neural Stem Cells for Cell-Based Medicinal Products

**DOI:** 10.3390/cells10092377

**Published:** 2021-09-09

**Authors:** Beatriz Fernandez-Muñoz, Ana Belen Garcia-Delgado, Blanca Arribas-Arribas, Rosario Sanchez-Pernaute

**Affiliations:** 1 Cellular Reprogramming and Production Unit, Andalusian Network for the Design and Translation of Advanced Therapies, 41092 Sevilla, Spain; anab.garcia.delgado@juntadeandalucia.es (A.B.G.-D.); blanca.arribas@juntadeandalucia.es (B.A.-A.); 2 Department of Pharmacy and Pharmaceutical Technology, School of Pharmacy, University of Sevilla, 41012 Sevilla, Spain

**Keywords:** neural progenitors, neural precursors, NSC, regenerative medicine, advanced therapies, central nervous system, safety, efficacy, scalability, quality control

## Abstract

Neural stem cells represent an attractive tool for the development of regenerative therapies and are being tested in clinical trials for several neurological disorders. Human neural stem cells can be isolated from the central nervous system or can be derived in vitro from pluripotent stem cells. Embryonic sources are ethically controversial and other sources are less well characterized and/or inefficient. Recently, isolation of NSC from the cerebrospinal fluid of patients with spina bifida and with intracerebroventricular hemorrhage has been reported. Direct reprogramming may become another alternative if genetic and phenotypic stability of the reprogrammed cells is ensured. Here, we discuss the advantages and disadvantages of available sources of neural stem cells for the production of cell-based therapies for clinical applications. We review available safety and efficacy clinical data and discuss scalability and quality control considerations for manufacturing clinical grade cell products for successful clinical application.

## 1. Neural Stem Cells

Neural stem cells (NSC) are self-renewing, multipotent cells that generate neurons and glial cells during development and maintain brain homeostasis. NSC can potentially migrate into damaged areas to promote functional and structural tissue repair. In addition, NSC have the capability to secrete trophic factors (e.g., glial cell-derived and brain-derived neurotrophic factors) that can stimulate endogenous repair mechanisms. Similar to mesenchymal stem cells (MSC), NSC can exert immunomodulatory effects and transplantation of NSC has been shown to inhibit T-cell proliferation. All these properties make NSC attractive for regenerative therapies, in particular considering the limited capacity for self-repair and the lack of effective therapies for most disorders of the central nervous system (CNS) [1,2,3].

Here, we discuss the main advantages and disadvantages of available and potential sources of human NSC, current evidence of their safety and efficacy, as well as scalability and quality control considerations for the development of clinical-grade NSC-based medicinal products.

**Figure 1:**
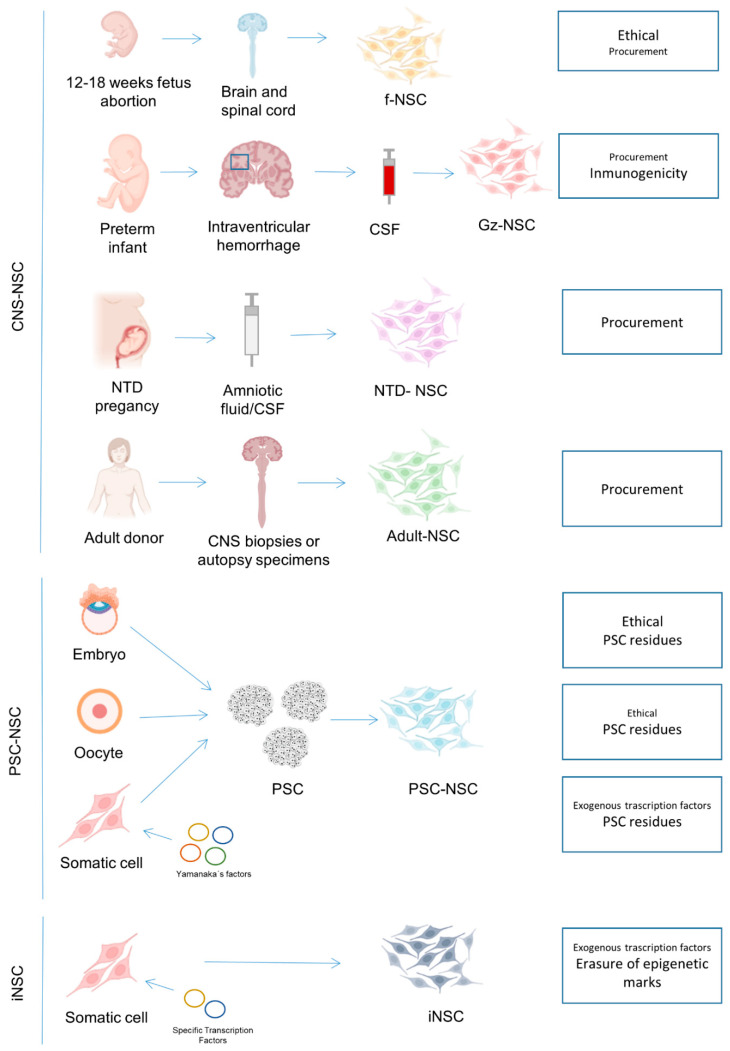
Available sources for NSC production and major concerns for each of them. CNS: central nervous system; PSC: pluripotent stem cells; fNSC: fetal neural stem cells, CSF: cerebrospinal fluid; Gz-NSC: germinal zone neural stem cells; NTD: neural tube defects; iPSC: induced pluripotent stem cells; ESC: embryonic stem cells; pESC: parthenogenetic stem cells; ntESC: nuclear transfer embryonic stem cells; iNSC: induced neural stem cells.

So far, most clinical trials have used fetal NSC (fNSC), but NSC from other sources such as pluripotent stem cells (PSC) are increasingly reaching the clinical stage. Furthermore, newer sources for primary NSC, such as the cerebrospinal fluid (CSF) of spina bifida or from premature infants with intracerebroventricular hemorrhage (ICVH) have been reported [4,5,6,7] and may become available in the near future (Figure 1).

NSC are characterized by the expression of typical markers such as NESTIN or SOX2 and the capability to generate all three neuroectodermal lineages [8]. There is some variability in the degree of stemness of NSC used in clinical trials, i.e., stem cells, progenitors, or precursors, as cells may not be fully synchronized, and these terms are sometimes used interchangeably. On the other hand, neural cells with restricted differentiation or proliferation potential, intended for the replacement of specific functional populations—such as oligodendrocyte precursor cells for remyelination, retinal pigmented epithelium for photoreceptor support, or dopamine neuroblasts for Parkinson disease—stand apart, and will not be reviewed here.

NSC derived from MSC, peripheral blood cells, or other somatic cell types by chemical transdifferentiation are being used in a few clinical trials, for example for spinal cord injury or stroke (reviewed in [9]). However, there is much debate about whether these cells are truly neural, since the demonstration of the generation of functional neurons from these cells is lacking and the mechanisms underlying the chemically induced transdifferentiation are largely unknown [9,10]. Therefore, we have not included transdifferentiated NSC in this review. Neither have we included NSC from the peripheral nervous system (PNS) that can be isolated from intestine, skin, olfactory bulb, and other tissues, because these are derived from the neural crest, express different markers, and generate a different progeny, including mesenchymal derivatives, and, therefore, represent a markedly different subtype of NSC [11].

## 2. Neural Stem Cells from the Central Nervous System

### 2.1. NSC Isolated from Human Fetal Neuroectoderm

Most clinical trials performed so far have employed NSC obtained from human fetal CNS (brain and/or spinal cord) (Table 1). Most often the tissue is collected from elective termination of pregnancies, which is a cause of major ethical concerns and opposed by many on moral and religious grounds [12]. The idea that the use of fetal tissue could encourage women to abort has been put forward by pro-life activists, although there is no evidence that using fetal tissue for therapeutic or commercial purposes increases the number of elected abortions [13]. Nevertheless, the use of fNSC is socially controversial and strictly regulated, being prohibited in some countries [14].

The use of fNSC from spontaneous abortions can partially bypass these issues, but these miscarriages are rare and unpredictable, so it is difficult to plan the procedures for an efficient scaling up. Furthermore, cells from spontaneously aborted fetuses could contain serious genetic defects requiring careful screening and selection before cell isolation (e.g., use only abortions caused by traumatisms). 

In contrast with early transplantation trials, in which fetal CNS tissue was transplanted into the patient soon after dissection to ensure viability, the optimization of in vitro culture of fNSC has solved many of the major supply and logistic hurdles of using fresh tissue [15,16]. On the other hand, including an in vitro cell expansion step, changes the regulatory classification of fNSC into a medicinal product (FDA/EMA), with ensuing requirements to comply with current good manufacturing practices (GMP) and other regulations.

**Table 1:** Clinical trials with neural stem cells.

Cell Type	Disease	ClinicalTrials.Gov Identifier	Phase	Location	Starting Year	Dose	Infusion Route	Principal Investigator	Manufacturer	References
fNSC	Multiple Sclerosis	NCT03269071	Phase 1	Italy	2017	0.7–5.7 million cells/kg	Intrathecal	Gianvito Martino	Stefano Verri Laboratory of cellular and gene therapies	Not found
fNSC	Multiple Sclerosis	NCT03282760	Phase 1	Italy/Switzerland	2017	5–24 million	Intracerebroventricular	Angelo L Vescovi	Laboratorio Cellule Staminali of Terni	Not found
fNSC	Amyotrophic Lateral Sclerosis	NCT01640067	Phase 1	Italy	2011	2.25–4.5 million (unilateral or bilateral)	Intraspinal	Angelo L Vescovi	Laboratorio Cellule Staminali of Terni	[16,17]
fNSC	Amyotrophic Lateral Sclerosis	NCT01730716	Phase 2	United States	2013	2–8 million (bilateral)	Intraspinal	Not detailed	Neuralstem Inc.	[18]
fNSC	Amyotrophic Lateral Sclerosis	NCT01348451	Phase 1	United States	2009	0.5–1 million (unilateral or bilateral)	Intraspinal	Not detailed	Neuralstem Inc.	[18,19,20,21,22]
fNSC	Neuronal Ceroid Lipofuscinosis	NCT01238315	Phase 1	United States	2010	500–1000 million	Intracerebral	Nathan Selden	StemCells, Inc.	Not found
fNSC	Neuronal Ceroid Lipofuscinosis	NCT00337636	Phase 1	United States	2006	500–1000 million	Intracerebral	Robert Steiner	StemCells, Inc.	[23]
fNSC	Parkinson Disease	NCT03128450	Phase 2/3	China	2017	4 million	Nasal	Jie Li	Shanghai Angecon Biotechnology Cooperate.	Not found
fNSC	Ischemic Stroke	NCT03296618	Phase 1	China	2012	12–80 million	Intracraneal	Xu Ruxiang	Neuralstem Inc. (currently Palisade Bio, Inc.)	[24,25]
fNSC	Spinal Cord Injury	NCT02688049	Phase 1/2	China	2016	10 million	Not detailed	Jianwu Dai,	Not detailed	Not found
fNSC	Spinal Cord Injury	NCT01772810	Phase 1	United States	2014	1.2 million (bilateral)	Intraspinal	Joseph Ciacci	Neuralstem Inc.	[26]
fNSC	Spinal Cord Injury	NCT02163876	Phase 2	United States/Canada	2014	Not detailed	Intraspinal	Stephen Huhn	StemCells, Inc.	[27,28]
fNSC	Spinal Cord Injury	NCT01725880	Phase 1/2	Switzerland	2012	Not detailed	Intraspinal	Stephen Huhn	StemCells, Inc.	[27,28]
fNSC	Spinal Cord Injury	NCT01321333	Phase 1/2	Switzerland/Canada	2011	Not detailed	Intraspinal	Stephen Huhn	StemCells, Inc.	[27,28]
fNSC	Spinal Cord Injury	NCT03069404	Phase 1/2	Switzerland/Canada	2017	Not detailed	Intraspinal	Armin Curt	StemCells, Inc.	[27,28]
fNSC	Ischemic Encephalopathy	NCT02854579	Not Applicable	China	2016	4 million	Intrathecal	Zuo Luan	Not detailed	[29]
fNSC	Age-Related Macular Degeneration	NCT01632527	Phase 1/2	United States	2012	0.3–1 million	Subretinal	Stephen Huhn	StemCells, Inc.	Not found
fNSC	Geographic atrophy age-related macular degeneration	NCT02467634	Phase 2	United States	2015	Not detailed	Subretinal	Joel Naor	StemCells, Inc.	Not found
fNSC	Geographic atrophy age-related macular degeneration	NCT02137915	Phase 1/2	United States	2014	Not detailed	Subretinal	David Birch	StemCells, Inc.	[30]
fNSC	Pelizaeus-Merzbacher Disease	NCT01005004	Phase 1	United States	2009	300 million	Intracerebral	Stephen Huhn	StemCells, Inc.	[31]
fNSC	Pelizaeus-Merzbacher Disease	NCT01391637	Phase 1	United States	2011	Not detailed	Not detailed	Stephen Huhn	StemCells, Inc.	Not found
fNSC	Retinitis Pigmentosa	NCT04284293	Phase 1	United States	2020	0.2–1 million	Subretinal	David Liao	Not detailed	Not found
fNSC?	Cerebral Palsy	NCT03005249	Not Applicable	China	2016	Not detailed	Not detailed	Jing Liu	Not detailed	Not found
fNSC expressing GDNF	Amyotrophic Lateral Sclerosis	NCT02943850	Phase 1	United States	2017	Not detailed	Intraspinal	Robert H. Baloh	Not detailed	Not found
fNSC v-myc inmortalized loaded with an oncolytic virus	Malignant Glioma	NCT03072134	Phase 1	United States	2017	Not detailed	Intracerebral	Maciej S Lesniak	Not detailed	[32]
fNSC v-myc immortalized NSC expressing CD	Glioma	NCT01172964	Phase 1	United States	2010	10–50 million	Intracerebral	Jana Portnow	City of Hope	[33]
fNSC v-myc immortalized expressing CD	Gliomas	NCT02015819	Phase 1	United States	2014	50–150 million	Intracerebral	Jana Portnow	City of Hope	[34]
fNSC v-myc inmortalized expressing CE	Gliomas	NCT02192359	Phase 1	United States	2016	Not detailed	Intracerebral	Jana L Portnow	Not detailed	Not found
fNSC v-myc inmortalized expressing CE	Glioma	NCT02055196	Phase 1	United States	2014	Not detailed	Intracerebral	Jana Portnow	Not detailed	Not found
fNSC c-myc inmortalized (inducible)	Ischemic Stroke	NCT03629275	Phase 2	United States	2018	20 million	Intracerebral	Richard Beckman	ReNeuron Limited	Not found
fNSC c-myc immortalized (inducible)	Stroke	NCT02117635	Phase 2	United Kingdom	2014	20 million	Intracerebral	Keith W Muir	ReNeuron Limited	[35]
fNSC c-myc immortalized (inducible)	Stroke	NCT01151124	Phase 1	United Kingdom	2010	2-million	Intracerebral	Not detailed	ReNeuron Limited	[36,37]
fNSC c-myc immortalized (inducible)	Peripheral Arterial Disease	NCT01916369	Phase 1	United Kingdom	2014	20–80 million	Intramuscular	Jill JF Belch	ReNeuron Limited	Not found
ESC-NSC	Spinal Cord Injury	NCT04812431	Phase 1/2	Republic of Korea	2021	Not detailed	Intrathecal	Dong Ah Shin	S.Biomedics Co., Ltd.	Not found
ESC-NSC	Ischemic Stroke	NCT04631406	Phase 1/2	United States	2021	Not detailed	Intracerebral	Gary K Steinberg	Not detailed	Not found
pPSC-NSC	Parkinson Disease	NCT02452723	Phase 1	Australia	2016	30–70 million	Intracerebral	Andrew Evans	International Stem Cell Corporation	[38]
iPSC-NSC	Parkinson Disease	NCT03815071	Phase 1	China	2019	Not detailed	Not detailed	Not detailed	Allife Medical Science and Technology Co., Ltd.	Not found

Terms “neural progenitor”, “neural precursor” and “neural stem cell” were used to look for clinical trials at ClinicalTrials.gov, by the US National Library of Medicine (https://clinicaltrials.gov/ last accessed on 14 May 2021). Molecular studies with no NSC transplant have been excluded. More differentiated cells derived from NSC such as oligodendrocyte precursor cells or interneuron progenitors have not been included in this review although it is worthy to note that these studies can be also relevant when designing an NSC therapy as the limit between NSC and more committed progenitors is difficult to stablish. Clinical cases or clinical trials registered in other databases such as Korean Clinical Research Information Service (https://cris.nih.go.kr/cris/info/introduce.do?search_lang=E&lang=E, accessed on 14 May 2021) have not been included in this table. fNSC: fetal neural stem cells; GDNF: Glial derivate neurotrophic factor; CD: cytosine deaminase; CE: carboxylesterase; ESC: embryonic stem cells; pESC: parthenogenetic stem cells; iPSC: induced pluripotent stem cells.

Clinical studies with fNSC have demonstrated some degree of efficacy in various conditions. For example, transplantation of fNSC in patients with cervical spinal cord injury resulted in the restoration of sensorimotor function [39]. Luan and colleagues described acceleration of motor development and functional improvements in children with severe cerebral palsy [40]. Motor improvements and better response to medication were reported in Parkinson patients, although the neuroimaging showed only transient effects and a steady decrease of Fluoro-DOPA putaminal uptake in the case shown in the publication [41]. Quiao and colleagues also reported improvements in neurological function after co-transplantation of fNSC and MSC in stroke patients [42]. At least three clinical trials conducted with the NSI-566 fNSC have reported some positive results in Amytrophic Lateral Sclerosis (ALS) and Stroke [18,24,25], although in our recent meta-analysis no significant improvement was found overall with fNSC in ALS [43]. Nittala and colleagues recently showed that transplantation of the fNSC line HuCNS-SC slows down lesion growth in age-related macular degeneration [30]. Many other clinical trials have shown modest, if any, efficacy results, with improvements often only seen in some patients [16,21,27,31,44]. Overall, the evidence for fNSC efficacy is mostly anecdotal, with no clinical trial demonstrating sustained recovery from the pathological condition.

Importantly, most trials do not include adequate controls because of the invasive nature of the delivery procedure, so it is complicated to establish the real effect of fNSC. For similar reasons, these phase I or phase I/II trials include severe, late-stage patients, as the main goal is to analyze safety and are not powered to detect efficacy. Another factor to take into account is that not all fNSC are equivalent, as they are obtained from different donors, at different developmental ages, and from different CNS regions, which can all have an impact on the outcome. Furthermore, some fNSC lines are isolated from the whole forebrain and/or spinal cord and are therefore a heterogeneous mixture of NSC with different region-specific phenotypes and may display different proliferation and differentiation potential according to the region where they originate (reviewed in [45]). Importantly, the biology of NSC and their relationship with the niche and with the immune system is still poorly understood, complicating the development of cell products targeting specific disease mechanisms, and the definition of optimal doses, route of administration, and immunosuppression requirements.

Although most clinical studies report only mild adverse effects, their immunogenic potential is a safety concern. fNSC have been shown to be poorly immunogenic with no expression of MHC-I and low levels of MHC-II, and cells can survive years after the cessation of immunosuppression [19,23,46]. However, other studies have shown that NSC can induce immune responses. Gupta and colleagues found that two out of four transplanted subjects developed donor-specific HLA alloantibodies, demonstrating that fNSC can elicit an immune response [31]. Furthermore, overexpression of MHC molecules has been described in the pro-inflammatory niche found in the graft site after transplantation [47]. Therefore, transplantation of fNSC has been often associated with long-term immunosuppression.

Tumor formation is a safety concern even if reports of graft overgrowth, tumor, or mass formation have been very rare, and most often related to fetal tissue grafts, for example in patients with Huntington’s disease [48,49]. There is a single report of donor-derived tumors in one Ataxia Telangiectasia patient grafted with expanded fNSC from multiple donors [50]. In this boy, the slow-growing tumors were well-differentiated and contained neurons and glial cells, including ependymal cells. It is very likely that the host environment facilitated the development of these nodules as patients with Ataxia Telangiectasia have an impaired immune response. Furthermore, although only karyotypically normal fetuses were used for isolation, testing of genetic stability by more precise methods such as comparative genomic hybridization was not performed at the end of the expansion stage. Safety studies should include genetic stability, growth factor dependency, and in vivo tumorigenesis assays. Particular care should be taken when grafting in permissive environments (young patients, neurogenic regions) as the niche is determinant in the growth and differentiation of the grafted NSC [49].

Some clinical trials have used genetically modified fNSC to increase their therapeutic potential. For example, taking advantage of the tumor tropism shown by NSC, some groups have genetically modified fNSC to manufacture anti-cancer therapies (mainly with cytosine deaminase and carboxylesterase) and the results from at least one clinical trial have demonstrated the safety and feasibility of this approach [34]. Immortalized NSC poses an increased risk of tumor formation and may impact the cellular response to the environment and therefore affect their safety and efficacy profiles. Hence, the efficacy and, especially, the safety profile of these cell products should be carefully assessed during the preclinical and clinical stages. Available data suggest, however, that some of these lines can be safe and efficacious. For example, Kalladka and colleagues showed that transplantation of the *C-MYC* conditionally immortalized CTX0E03 NSC line improved neurological function after ischemic stroke, with no report of cell-related serious adverse events [36,37].

### 2.2. NSC Isolated from the Cerebrospinal Fluid

We and others have recently reported that primary NSC can be isolated from the CSF, collected for diagnostic or therapeutic purposes, of infants diagnosed with severe intracerebroventricular hemorrhage (IVH) or neural tube defects (NTD) [4,5,6,7,51].

Severe IVH is a common complication of preterm infants that entails the rupture of the germinal zones of the ganglionic eminences where NSC resides. This rupture entails the shedding of NSC to the ventricular space. Ventricular neuroendoscopy is performed in these patients as a therapeutic approach to reduce the intracranial pressure and the associated deleterious effects of the high intracranial pressure and of the blood degradation products. We have recently demonstrated that NSC can be easily retrieved from the collected CSF and the cells can be effectively expanded and banked and are not tumorigenic [4]. As these CSF samples are usually discarded, the isolation of CSF-derived NSC does not raise special ethical concerns. These NSC, although similar to fNSC derived from abortions, display distinctive hallmarks related to their regional and developmental origin in the germinal zone of the ventral forebrain (ganglionic eminences) so we have called them germinal zone (Gz)-NSC. The Gz-NSC express markers of neural stem cells at high levels, comparable to fNSC (CD133, NESTIN, SOX2, BLBP) [4]. One of the main differences is that while forebrain fNSC are obtained from an early gestational age (usually before 16 weeks) and generate mostly dorsal (PAX6 positive) phenotypes, Gz-NSC are isolated from premature infants at a later gestational age (26–32 weeks), have a ventral specification (OTX2 and NKX2.1 positive) and participate in late-stage corticogenesis, generating interneurons and oligodendrocytes [52], so they might be useful for diseases in which there are deficient or dysfunctional interneurons or myelination, such as cerebral palsy or schizophrenia. Intracerebral transplantation of Gz-NSC in nude mice resulted in small neural grafts with no tumor formation or adverse reaction. Further safety studies have to be carried out to ensure that Gz-NSC does not pose a risk of tumor formation and to determine the optimal cell dose, route, etc. In addition, the high expression of MHC class II molecules in Gz-NSC [4] can impede their allogenic use. The in vitro and in vivo differentiation potential of Gz-NSC is promising, but further safety and efficacy experiments are required to determine whether Gz-NSC will be useful in a clinical setting.

Neural tube defects (NTD) are caused by an incomplete closure of the neural tube during embryonic development [53]. Due to the incomplete closure of the neural tube, NSC are shed into the CSF and this CSF is released into the amniotic fluid that can be collected during intra-utero surgeries or diagnostic procedures. Several studies have demonstrated the presence of NSC in CSF or amniotic fluid of NTD patients and not in healthy donors. These NSC expressed typical markers and could differentiate into the three neural lineages, and mediated functional recovery when transplanted in stroke or NTD animal models [5,6,7,51]. These cells could be relevant for the development of cell therapy-based approaches especially for patients diagnosed with NTD, but few animal experiments have been done to demonstrate their safety. Furthermore, a screening of NTD-associated mutations should be performed in the NSC lines derived from NTD patients prior to their use for cell therapy.

The isolation of NSC from CSF offers access to perinatal NSC—as they come from CSF of preterm infants and intra-utero surgeries—without the ethical concerns associated with the use of fetal tissue from abortions. They are isolated from CSF samples retrieved for diagnostic or therapeutic purposes where only part of the sample is used and most of the content is discarded. However, isolation from these CSF sources is a recent discovery so there is no clinical experience and limited data from animal experiments. The scarcity of starting material is another inconvenience, as these are rare disorders and so far we have failed to obtain NSC from CSF samples from obstructive hydrocephalus, a much more frequent pathology [4]. However, we have successfully expanded and froze Gz-NSC for banking, opening up the possibility to use them in future clinical trials.

### 2.3. NSC Isolated from Biopsy and Autopsy Material

NSC can be also isolated from adult and fetal CNS biopsies or autopsy specimens. They have been isolated from different brain regions including the cortex, subventricular zone, hippocampus, midbrain, and spinal cord [54,55,56]. Isolation from ultrasonic aspiration during epilepsy surgery has also been reported [57]. Scarcity of starting material and few NSC in the adult tissue are principal drawbacks that have hampered the development of cell products based on these cells. However, a single case study reported the isolation of NSC from adult brain biopsies (prefrontal cortical and subcortical region) and transplantation into the basal ganglia of Parkinson’s patients producing a long-lasting motor improvement [58]. Based on these results, a clinical trial to better study the safety of the therapy is being conducted (NCT03309514).

## 3. NSC Derived from Pluripotent Stem Cells

NSC can be also obtained by differentiation of pluripotent stem cells (PSC). Human PSC can be derived from the inner cell mass of blastocyst-stage embryos (embryonic stem cells, ESC), obtained by chemical activation (parthenogenetic stem cells, pESC) or nuclear transfer (ntESC) of unfertilized oocytes or generated by epigenetic reprogramming of somatic cells with a defined set of transcription factors (induced pluripotent stem cells, iPSC) (Figure 1).

Since ESC are derived from embryos, usually from surplus embryos of in vitro fertilization procedures, the generation and use of ESC is ethically unacceptable for many. For some cultures and religions, life begins at fertilization and they opposed the destruction of embryos for any purpose—regardless of whether they are produced by natural means, by in vitro fertilization, or by nuclear transfer—and regardless of the embryo developmental stage [59]. For this reason, some countries prohibit the use of embryos and ESC, although, in some countries where ESC derivation is forbidden, their use in research is allowed. Even in countries where it is allowed to use embryos up to day 14 of development, stringent ethical committees and oversight panels regulate the use of these cells, limiting their use for regenerative purposes [14].

Although the generation of pESC and ntESC circumvents the use of embryos, the use of human oocytes is also a cause of ethical concern. Many consider the process of collecting oocytes from normal females unethical since it is non-beneficial for them, it is painful, potentially risky for their general health (requires surgery and hormonal treatment), and especially for their reproductive health (their reproduction success can be compromised because fewer oocytes are available for reproductive purposes) [12,59]. Furthermore, the generation of ntESC implies the creation of an embryo exclusively for research purposes, a fact that is viewed as ethically unacceptable for many [12].

iPSC overcome the ethical problems associated with ESC, ntESC, and pESC as they are generated from somatic cells, usually fibroblasts or blood cells obtained by non or minimally invasive procedures, avoiding the use of embryos or oocytes. However, the extensive molecular manipulation during reprogramming and the artificial conditions of in vitro neural induction may limit their utility for regenerative purposes. Thus, the safety and potency profile of the resulting NSC product should be carefully characterized during the pre-clinical stage. There are multiple reports informing on the substantial differences between iPSC and ESC, including different gene expression signatures, residual retention of transcriptional and epigenetic memory of the somatic cell of origin, the existence of lab-specific gene expression differences, early senescence of iPSC progeny, and single-cell heterogeneity of iPSC (reviewed in [60]). Other logistic and safety issues with iPSC are the low reprogramming efficiency, tumorigenesis related to insertional mutagenesis, and to the fact that reprogramming enriches mutations in oncogenes [61], and the propensity of cell lines to give rise to derivatives from a specific germ layer [62]. Nevertheless, many of these reprogramming issues are more theoretical than practical and may not be relevant for iPSC-NSC clinical safety [13]. In fact, clinical trials using iPSC-NSC are underway (Table 1).

Notwithstanding, the use of ESC is considered safer for the patient by some researchers because ESC are truly pluripotent, blastocyst-derived cells, that better recapitulate the processes of natural embryogenesis and differentiation [59], leading to the generation of functional somatic cell types with less risk of mutagenesis or transformation. Consequently, despite the ethical drawbacks, the production of ESCs and ESC-derived NSC is still being pursued, with several ongoing clinical trials (Table 1).

Although at first iPSC were envisioned to be used in an autologous setting, soon researchers realized that the generation of iPSC under GMP conditions (required for clinical use) for a single patient, is time-consuming, expensive, and hardly viable, and therefore, most clinical trials with iPSC-derived products are being performed in an allogeneic setting [63]. The immunological reaction triggered by allogeneic iPSC-NSC can be overcome by co-treatment with immunosuppressant drugs, use of HLA-matched iPSC or HLA ablated iPSC by gene-editing technologies such as CRISPR (reviewed in [64,65]). To facilitate the search for compatible donors and foster the development of cell therapies with iPSC-derived products, iPSC banks with common HLA haplotypes are being created at different locations such as Japan [66], the United Kingdom [67], or the Republic of Korea [68]. 

The use of PSC to obtain NSC for cell-based therapies could foster the development of new products. Whether the ideal PSC source—i.e., technically feasible, safe, and efficacious, and socially acceptable—will end up to be ESC, pESC, or iPSC remains to be seen.

A common safety problem of the clinical use of NSC derived from any PSC is the potential presence in the final product of contaminant pluripotent cells that can form teratomas or other tissues. Undifferentiated PSC develop tumors in the brain in experimental settings [69,70] and several groups have reported teratoma or overgrowth formation after PSC-NSC transplantation in several disease models [71,72,73,74]. Therefore, caution should be taken to not transplant residual PSC. So far, clinical trials with carefully controlled PSC-derived products have shown a lack of residual undifferentiated PSC and negligible tumorigenic potential of NSC [75].

Another shared problem for all PSC is the fact that differentiation of NSC from PSC requires tedious manipulations that might compromise the quality of the generated cells and increase the costs and time of the manufacturing process. In contrast, primary NSC do not need differentiation procedures (usually only expansion) and better reproduce terminal differentiation and the functional characteristics of the cells naturally present in the brain. Protocols used to generate NSC from PSC do not completely reproduce the brain physiological niche (matrix, cell interactions, physical forces, etc.), and therefore, the resulting NSC do not fully resemble the “authentic” NSC, neither captures all their potential. More realistic induction protocols by using bioengineering with positional information, mechano-geometrical inputs, mimicking the signaling input of extra-CNS tissues (meninges, vasculature, etc.), specific extracellular matrix, differentiation in 3D, etc. are being developed and probably will bring a new generation of more “authentic” and therapeutically potent PSC-derived NSC [76,77].

An additional problem with PSC is that many of the cell lines available for research do not have appropriate informed consent and may not be eligible for clinical research or potential commercialization [12]. Furthermore, PSC-NSC manufacturing requires a long and complex manufacturing process that requires trained operators. Additionally, manufacturing implies the banking of the intermediate cell product (PSC cells) whose quality and safety have to be carefully controlled. Moreover, PSC culture is more difficult to scale up than that of primary NSC, as PSC grow as colonies and have stringent culture conditions (special matrices, growth factors, etc.). Although some bioreactors are being developed to scale up PSC and NSC cultures [78,79], in general, PSC-derived products are more difficult and expensive to scale up and to achieve GMP compatibility than the primary lines. The costs can be decreased by using the same PSC line for different clinical uses. For example, cell products derived from the hESC GMP line MA09 have been used in 11 different clinical trials. Many of those GMP PSC lines used in clinical trials were first established as laboratory-grade lines and converted into clinical-grade lines afterward [63]. The use of these GMP adapted lines for multiple trials would decrease time and costs while allowing standardization and exchange of knowledge. In this sense, the development of GMP banks of PSC will foster the development of new therapies.

Although data from animal models suggest that PSC-NSC can be safe and efficacious [9], clinical data with PSC-derived NSC are still limited, but several clinical trials are underway (Table 1) and will probably clarify whether the transplantation of PSC-NSC in humans is safe and feasible.

## 4. Induced NSC

Induction of direct reprogramming from somatic cell types to NSC (iNSC), without passing through a pluripotent state, can be achieved by employing specific transcription factors (TF) and sometimes with the aid of pharmacological compounds. This process causes a shift in the transcriptomic profile of a given cell type and the acquisition of the transcriptional profile of another cell type, losing its own phenotype. The initial study followed Yamanaka’s strategy to reprogram fibroblasts directly into neurons [80]. This provided a new strategy to obtain NSC using forced exogenous gene expression, but the resultant neural cells divided only a few times. In two parallel studies [81,82] stably proliferating iNSC were generated by time-restricted expression of *OCT4* during the first days of reprogramming. Based on *OCT4* and other TF and supplements, several studies have reported the generation of iNSC from different cell sources such as fibroblasts and peripheral blood cells [83,84,85,86,87]. Additionally, *SOX2* alone or in combination with other TF has been demonstrated to be sufficient for the direct conversion of somatic cells into iNSC. *SOX2* is the most frequently employed factor in the second-generation reprogramming protocols, given its function as a master regulator for the specification and maintenance of progenitors of the neural tube and the CNS [88,89,90,91]. We used non-integrative (Sendai virus) overexpression of *SOX2* and *c-MYC* to obtain NSC from cord blood CD133^+^ cells [92]. As with reprogramming to pluripotency, starting with a cell type that has few age-related epigenetic marks and mutations, like cord blood stem cells, is expected to result in a safer cell product. Additionally, the use of non-integrative approaches is preferable. Indeed, the use of vectors with polycistronic cassettes that give rise to fusion proteins that do not physiologically exist in the cells can lead to uncontrolled and undesired effects [93]. Furthermore, TF used to induce direct conversion can bind to gene regulatory regions of genes unrelated to the molecular nature of the desired cell [94]. This is also true in some cases for iPSC generation [95] and care should be taken to control safety issues associated with the use of TF, in particular when they are in polycistronic cassettes. Several studies proposing the use of safer non-viral vectors and chemical-based protocols for direct conversion of somatic cells into iNSC suggest that small molecules can increase the efficiency of iNSC generation [96,97,98].

iNSC have been transplanted in animal models of spinal cord injury, stroke, Parkinson’s Disease, or multiple sclerosis, among others [99]. Results from these studies suggest that iNSC can ameliorate pathological conditions and increase survival showing no tumor formation potential. However, to date, no clinical data of iNSC transplantation are yet available.

Direct reprogramming avoids cumbersome iPSC generation and differentiation procedures decreasing time and costs and minimizing the risk of having residues of tumorigenic cells in the final cell product. However, during direct reprogramming with TF, the epigenetic memory of the parental cell line is not erased, and this could lead to the reversion to the parental phenotype in some contexts. This means that directly converted cells may not attain a durable terminal phenotype [94]. We used electrophysiology, showing the presence of evoked action potentials and the response to neurotransmitter agonists and antagonists to confirm neuronal differentiation of iNSC [92]. We believe the phenotypic and functional assessment is mandatory to demonstrate a real lineage fate conversion. Although iPSC also have the problem of residual epigenetic memory, during reprogramming to pluripotency there is a step of epigenetic erasing that considerably decreases parental marks. Another drawback of iNSC is the fact that most protocols for direct reprogramming show low efficiency and/or purity and the mechanisms underlying direct reprogramming are not completely unraveled. Therefore, iNSC are not yet considered optimal for cell therapy applications.

## 5. Clinical Grade NSC Therapies: Safety, Efficacy, Scalability and Quality Control Considerations 

The main safety risks that patients treated with NSC products can be exposed to are related to delivery procedures; allergic or immune reactions to the medicinal product (NSC or other reagents used during manufacturing); tumor formation; heterotopic neuronal differentiation with functional disruption, and microbiological or viral contamination. All these risks should be minimized by in vivo/in vitro extensive assessment during the pre-clinical stages. The benefits have to overcome the risks. Safety has been extensively proven for fNSC in clinical trials. With only a few clinical trials with PSC-NSC and few results published from these clinical trials, their safety has been only confirmed in animal models, with some reports describing tumor formation risks [74,100,101,102]. Other sources, such as CSF-derived NSC and iNSC are not yet being tested in clinical trials (Table 2).

Most clinical trials transplant the NSC into the CNS parenchyma, intracerebrally or intramedullary. Intravascular or intraventricular delivery could be appropriate for multifocal or widespread CNS diseases and might be safer, but animal data indicate that these routes result in poor cell engraftment [103] and can lead to heterotopic grafts [104], therefore, they are not commonly tested in clinical trials. Interestingly, there is one clinical trial studying nasal delivery of NSC (NCT03128450, Table 1). NSC transplantation shortly after CNS damage seems to be the most appropriate approach for several diseases since shortly after the injury the presence of molecules that promote cell growth predominates over that of molecules that oppose plasticity [105,106,107].

**Table 2:** Safety, efficacy, scalability, and quality control considerations for clinical application of NSC from different sources.

Origin	Type of NSC	Safety	Efficacy	Immunogenicity	Ethical Concerns	Procurement	Logistics/Scalability	References
CNS	fNSC	Safe as confirmed by multiple clinical trials and animal models	Some clinical studies describe positive results but none demonstrating sustained recovery	Immunosuppression required (only allogeneic setting is feasible).	Derived from human fetuses, usually from elected abortions	Difficult. Ethical concerns complicate procurement being forbidden in some countries	Easy. Once obtained, cells are easy to grow and scale-up	[16,17,18,19,20,21,22,23,24,25,26,27,28,29,30,31,32,33,34,35,36,37,38,39,40,42,50,108]
	Gz-NSC	Only confirmed in animal models	Not confirmed	No Immunosuppression required in autologous settings. High HLA expression can impede their allogenic use	No ethical issues as the original source (CSF of IVH preterm infants) is usually discarded and do not imply an extra surgery in the patient	Difficult. Few IVH cases and not all hospitals perform therapeutic neuroendoscopies to remove CSF in IVH patients.	Easy. Once obtained cells are easy to grow and scale-up. Autologous setting is costly and allogenic setting not possible due to the high HLA expression	[4]
	NTD-NSC	Only confirmed in animal models	Only confirmed in animal models	No Immunosuppression required in autologous settings. Immunosuppression required in allogenic treatments.	No ethical issues as the original source (CSF of NTD patients) is usually discarded and do not imply an extra surgery in the patient	Difficult. Few NTD cases	Easy. Cells are easy to grow and scale-up	[5,6,7,51]
	NSC from adult biopsies/autopsies	Safety confirmed in animal models and few clinical cases	Efficacy confirmed in animal models and few clinical cases	No Immunosuppression required in autologous settings. Immunosuppression required in allogenic treatments.	Biopsy procurement potentially risky for the patient	Difficult. Few cells in the source	Difficult. Few cells with probably lower growth capabilities.	[54,55,56,57,58]
PSC	iPSC-NSC	Only confirmed in animal models. Safety concerns related to the use of exogenous transcription factors, the enrichment of somatic mutations, and the fact that epigenetic marks from the original somatic cell are not totally erased. Turmorigenic risk due to potential PSC residues.	Only confirmed in animal models	No Immunosuppression required in autologous settings. Immunosuppression required in allogenic treatments.	Invasive surgery in some cases to obtain the initial cell type used for reprogramming (e.g., skin fibroblast isolation)	Easy. The initial cell type used for reprogramming is usually easily accessible e.g., coming from a skin biopsy or blood sample	Easy. Establishment of iPSC lines is time-consuming and requires trained operators but once the PSC cells are obtained they are easy to grow, allowing safe production of many cell doses.	[60,61,62,64,65,66,67,68,71,74,95]
	ESC-NSC	Only confirmed in animal models. Turmorigenic risk due to potential PSC residues.	Only confirmed in animal models	Immunosuppression required (only allogeneic setting is feasible).	Derived from human embryos	Difficult. Ethical concerns complicate procurement being forbidden in some countries	Easy. Establishment of ESC lines is time-consuming and requires trained operators but once the PSC cells are obtained they are easy to grow, allowing safe production of many cell doses.	[60,69,70,72,73,101,102]
	pESC-NSC	Only confirmed in animal models. Turmorigenic risk due to potential PSC residues. Haploid cells	Only confirmed in animal models	Immunosuppression required (only allogeneic setting is feasible).	Derived from human oocytes (ethical concerns about the payment to oocyte donors, the medical risks of oocyte retrieval, and with the affectation of their reproduction success as this can be compromised because fewer oocytes are available for reproductive purposes)	Difficult. Ethical concerns complicate procurement.	Easy. Establishment of pESC lines is time-consuming and requires trained operators but once the PSC cells are obtained they are easy to grow, allowing safe production of many cell doses.	[75,109]
	ntESC-NSC	Only confirmed in animal models. Turmorigenic risk due to potential PSC residues	Only confirmed in animal models	Immunosuppression required (only allogeneic setting is feasible).	Derived from human oocytes (ethical concerns about the payment to oocyte donors, the medical risks of oocyte retrieval, and with the affectation of their reproduction success as this can be compromised because fewer oocytes are available for reproductive purposes)	Difficult. Ethical concerns complicate procurement.	Easy. Establishment of ntESC lines is time consuming and requires trained operators but once the PSC cells are obtained they are easy to grow, allowing safe production of many cell doses.	[110]
Direct reprogramming	iNSC	Only confirmed in animal models. Safety concerns related to the use of exogenous transcription factors and the fact that epigenetic marks from the original somatic cell are not erased.	Only confirmed in animal models	No immunosuppression required in autologous settings. Immunosuppression required in allogenic treatments.	Invasive surgery in some cases to obtain the initial cell type used for reprogramming (e.g., skin fibroblast isolation)	Easy. The initial cell type used for reprogramming is usually easily accessible e.g., coming from a skin biopsy or blood sample	Easy. Establishment of iNSC lines is time-consuming but, once the iNSC are obtained, they are normally easy to grow, allowing safe production of many cell doses.	[80,81,82,83,84,85,86,87,88,89,90,91,92,93,94,96,97,98,99]

CNS: central nervous system. fNSC: fetal neural stem cells, Gz-NSC: germinal zone neural stem cells; NTD: neural tube defects; iNSC: induced neural stem cells; iPSC: induced pluripotent stem cells; ESC: embryonic stem cells; pESC: parthenogenetic stem cells; ntESC: nuclear transfer embryonic stem cells; IVH: intraventricular hemorrhage; CSF: cerebrospinal fluid; HLA: human leukocyte antigen.

Although immune reactions to transplanted NSC could be decreased by the use of autologous cells, autologous therapies are costly and difficult to scale up. As a matter of fact, no clinical trial is being performed with autologous NSC (Table 1). Although the CNS is considered immune-privileged, the allogeneic approach involves the risk of immune reactions in the donor and may require the use of immunosuppressive therapy (most commonly cyclosporine or tacrolimus). In this sense, it is worth mentioning that in clinical trials using NSC, few adverse events related to immune reactions have been reported. However, the actual need and optimal protocols of immunosuppression for allogenic NSC transplantation have yet to be established in humans [111].

NSC have the advantage over post-mitotic cells of being more plastic and expandable, being easier to scale up. The fact that their final fate and behavior will be dictated by the host environment is also relevant, but less amenable to control. For instance, a senescent niche could turn the transplanted cells senescent, even when derived from young donors [112,113]. On the other hand, a permissive niche could favor the formation of tumors as reported for fNSC in a case of Ataxia Telangiectasia [50]. This is even more relevant for PSC-NSC due to the intrinsic tumorigenic potential of PSC. Therefore, cell dose should be precisely determined during the pre-clinical stage to avoid safety issues. Furthermore, NSC that undergo long-term in vitro expansion tend to gain genetic mutations and lose the codes of transcription factors that determine positional identity [114]. Consequently, early passage NSC are preferable for clinical use, and phenotypic and genetic stability should be carefully controlled at the end of the expansion process.

Although safety has been extensively studied for fNSC with only minor adverse events described, efficacy results have been modest, as mentioned earlier, indicating that efforts have to be done to maximize the potency of NSC therapeutics. The use of scaffolds and co-treatments to reinforce the NSC effect is being investigated. For example, the use of drugs, neurotrophic factors, or other cells (mainly MSC) to modify the pro-inflammatory environment and to increase efficacy is being studied [115,116]. This approach appears reasonable since pro-inflammatory cytokines inhibit differentiation, proliferation, and, probably, functional integration of NSC progeny [117]. However, we still need to better understand how NSC exert their effect and interact with the immune system and how to produce the desired cell type in vivo, in order to favor integration and production of neuroprotective molecules in a timely manner to achieve efficacy and avoid unwanted side effects.

Available data regarding manufacturing details and quality control tests for the release of NSC-based products is rather scarce. Cell manufacturing should be performed according to the principles of GMP [118]. NSC are usually expanded and cryopreserved to generate master and working cell banks that should be properly characterized. The final cell product can be released as a ready-to-use product or as an intermediate product, to be reconstituted at the clinical site. In principle, a ready-to-use product is desirable to properly control every stage of cell manipulation, making it comply with GMP guidelines. Optimal conditioning solutions should be used to resuspend NSC. Different studies point to Pluronic and Hypothermosol as good options [119,120,121]. The use of fresh live products is a limitation for scaling-up and distribution, therefore, stable products that could be used “off-the-shelf” are needed. Transplantation of cryopreserved NSC-derived products is being tested [122] but more data are needed to demonstrate that cryopreserved NSC are functional and effective. Storage conditions should be selected to preserve the potency of the product for the longest period of time. Stability studies should be performed to assure that the cell product is stable within the specified shelf-life.

One of the problems when manufacturing NSC-derived products is the heterogeneity of the obtained cell population that can lead to variability in clinical outcomes. Fluorescence-activated cell sorting (FACS) has been used to prospectively isolate CD133^+^ NSC from the fetal brain under non-GMP conditions [123]. The use of magnetic separation systems or closed GMP compliant cell sorting devices could be interesting options to decrease lot-to-lot variability and obtain a purer cell product. Separation techniques could also be used to get rid of undesired cells (e.g., undifferentiated PSC). However, the inclusion of a purification step considerably increases the manufacturing costs.

When manufacturing cell-based products, materials and reagents should be selected for GMP compatibility, ideally, they should be xeno-free and defined, to avoid batch-to-batch variability and prevent the transmission of prions and adventitious viruses and immunologic reactions [124]. This sometimes requires changing the reagents used during the pre-clinical/basic research stage. Pre-clinical testing should ideally be performed with GMP or GMP-like lines as it has been shown that alterations in cell potency can arise due to subtle differences in reagents and manufacturing settings. The efficacy and safety studies should be carried out with the GMP product’s final formulation to avoid changes in important attributes [125,126,127].

NSC can be expanded in suspension as neurospheres or in adhesion. The neurosphere culture is the conventional approach for expanding NSC because it is considered to be favorable to maintain stemness [128]. However, neurosphere culture has some limitations, such as limited nutrition and oxygen penetration in the center of the neurosphere and positional heterogeneity of cells. Therefore, expansion in adhesion has been optimized to increase growth rates and cell homogeneity [129]. Defined matrices, such as laminin or fibronectin are preferable for GMP manufacturing, but they reduce the complexity of the extracellular environment and do not resemble the physiological NSC niche. The in vitro conditions selected to expand NSC should mimic the physiological conditions in order not to alter their functional and molecular properties [114].

Aseptic processing is a requisite to avoid contamination of cell products with microbes or physical/chemical agents that can alter potency and safety. The validation of the aseptic process (media fill) is a requirement for manufacturing GMP NSC-based products [130]. A documentary system according to GMP with standard operating procedures, specifications for all the products used for manufacturing, and detailed records is also a requirement for clinical development and GMP compliance.

Unfortunately, there is scarce information about applied quality control and testing regimens for the release of NSC-based products for clinical use. Quality controls should be selected case by case using a risk-based-approach analysis and taking into account FDA/USP or EMA/Ph. Eur. Guidelines, but most common quality control tests for release are described in Table 3. Many of these quality control tests should be performed to demonstrate the absence of microorganism or microorganism’s residues (e.g., endotoxins) in the final product. Special considerations should be taken for virus contamination especially in the COVID19 pandemic context [131]. They should be performed in addition to the aseptic processing that should be demonstrated for each batch production process by air-borne particle and microbiological monitoring techniques [132,133].

Ideally, one cell batch should be used for all the patients in a clinical study, but this is sometimes impossible, so measures should be taken to minimize lot-lo-lot variability and stringent controls should be performed in each lot to assure comparability and minimum quality. Having big lots decreases variability in clinical outcomes and decreases manufacturing costs.

Markers used to establish cell identity vary from one manufacturer to another, sometimes including only one marker (commonly NESTIN or CD133). However, more complete identity and purity tests, including markers for undesired cell types that can contaminate the final product, are highly recommended to recognize all cell types present in the final product and ensure consistency between lots. This is even more relevant when NSC are derived from PSC, to verify the lack of pluripotent cells in the final product. Acceptance limits should be established based on previous experience. For example, the identity release criteria for Neuralstem Inc. are: >99% NESTIN, 0% GFAP (glial fibrillary acidic protein-positive, astrocyte marker), 0% NG2 (oligodendroglial marker), 0% TUJ1 (BIII tubulin, neuronal marker), 0% IBA1 (microglial marker), 0% SMA (smooth muscle actin, a marker of dorsal root ganglia-derived cells), 0% VE-cadherin (a marker of vascular endothelial cells) [21,22]. Flow cytometry and qPCR are used to ensure the absence of pluripotent stem cells in the final product. For example [75], for parthenogenetic PSC derived NSC, Garitaonandia and coworkers showed by qPCR expression of *POU5F1* (OCT4) to be less than 0.01% (1 in 100,000 cells), (equivalent to that in fibroblasts) and by flow cytometry 0% OCT4 and 0% SSEA, with high expression (>95%) of neural progenitor markers (NES, MSI1, and SOX2).

The potency test is a key issue for the development of truly effective drugs. This test is a quantitative measure of biological or therapeutic activity, intended as an indication of efficacy. The main problem is that an understanding of the mechanism of action is required to define the potency assay, and, in many cases, it is not clear whether the therapeutic effect of NSC is based on their potential for differentiation and/or the release of trophic and other factors [117]. NSC secrete significant quantities of several neurotrophins such as nerve growth factor, brain-derived neurotrophic factor, and glial cell line-derived neurotrophic factor. The neuroprotective effect of NSC is supported by the presence of increased levels of these growth factors, usually without significant differentiation of the grafted NSC, suggesting that functional recovery is more linked to a trophic effect than a true cell replacement (reviewed in [117,147]). The NSC trophic effect has been mainly demonstrated in rodents, while a study characterizing the neurotrophic factor profile secreted by human NSC isolated from different sources and its relationship with efficacy is still lacking. Some manufacturers of NSC-based products use the tri-lineage differentiation as proof of potency, but if pre-clinical (and clinical) data indicate a predominant trophic effect, that potency test is useless and should be replaced by trophic factor quantification, establishing a range for product specification and comparability of batches. The most likely mechanism of action should be investigated during the pre-clinical stage; this will allow the design of an adequate potency test that ensures the functionality of the NSC product in the clinical phases [148]. Ensuring cell product potency and producing bona fide cell therapy products is key, especially considering the emergence of multiple producers offering fraudulent therapies based on scientifically un-sustained claims [149,150].

## 6. Conclusions

The therapeutic potential of NSC is broadly accepted by the scientific community but results from the clinical studies performed so far indicate that further studies are needed in order to improve their efficacy. Embryonic sources are controversial and strictly regulated, hampering advances. iPSC-derived NSC are seen as a promising source for the development of cell therapies because they do not pose ethical problems and are a scalable and “inexhaustible” source. However, the in vitro generated PSC-NSC, although similar to the primary cell lines do not truly recapitulate the phenotype and function of endogenous NSC and entail a risk of tumor formation. In addition, the reprogramming and differentiation processes are lengthy and costly under GMP conditions. Newer NSC sources and a better understanding of the neurobiology of the cells will surely help in the ambitious quest to repair the brain using cell-based therapies.

## Figures and Tables

**Table 3:** Common quality control tests performed to release NSC-based products.

Test	Method	Comments
Sterility	Turbidity testing after growth in TSB and TG media (Ph. Eur.2.6.1) [134] or media for automated detection systems (Ph. Eur. 2.6.27) [135]	NA
Mycoplasma	PCR, culture (Ph. Eur. 2.6.7) [136]	NA
Endotoxins	LAL assay (Ph. Eur. 2.6.14) [137]	NA
Adventitious viruses	PCR, cytopathic effect, others (Ph. Eur. 5.2.3 and Ph. Eur. 5.1.7) [138,139]	The viruses to detect should be evaluated case by case by risk analysis (Ph. Eur. 5.1.7) [139]
Cell number and Population doublings	Haematocytometer, automated cell counters, flow cytometer (Ph. Eur. 2.7.29) [140]	NA
Viability	Trypan blue, Calcein/Ethidium, 7AAD, PI (Ph. Eur. 2.7.29) [140]	This should be higher than 70% when transplanted [141]
Identity/Purity	Flow cytometry (Ph. Eur. 2.7.24) [142], PCR (Ph. Eur. 2.6.21) [143], immunofluorescence (Ph. Eur. 2.7.1) [144], others (Ph. Eur. 5.2.3) [138].	Markers of the possible contaminant cells (e.g., PSC) should be included [145]
Potency	Tri-lineage differentiation potential, expression of neurotrophic factors, etc. (Ph. Eur. 2.6.34) [146]	NA
Tumorigenicity	Karyotype, CGH array, trophic factor dependence, colony-forming assays in softagar, in vivo tumor formation assay in athymic mice (Ph. Eur. 5.2.3) [138]	NA
DNA Fingerprint	STR, VNTR (Ph. Eur. 5.2.3) [138]	NA

Selected European Pharamacopea (Ph. Eur.) chapters or guidelines that can be used as a reference have been included. TSB: Trypticase soy broth; TG: Thioglycollate; LAL: Limulus amebocyte lysate; PCR: Polymerase chain reaction; 7AAD: 7-Aminoactinomycin D; PI: Propidium iodide; CGH: Comparative Genomic Hybridization; STR: short tandem repeats; VNTR: variable number of tandem repeats; NA: not applicable.

## Data Availability

Data sharing is not applicable to this article.
